# Therapeutic effect of oral quercetin in hamsters infected with *Leishmania Viannia braziliensis*

**DOI:** 10.3389/fcimb.2022.1059168

**Published:** 2023-01-13

**Authors:** Rosiane Freire dos Santos, Thayssa Da Silva, Andréia Carolinne de Souza Brito, Job Domingos Inácio, Bianca Domingues Ventura, Michely Aparecida Polido Mendes, Bruno Fonseca Azevedo, Larissa Moreira Siqueira, Elmo Eduardo Almeida-Amaral, Patrícia Maria Lourenço Dutra, Silvia Amaral Gonçalves Da-Silva

**Affiliations:** ^1^ Laboratório de Imunofarmacologia Parasitária, Disciplina de Parasitologia/Faculdade de Ciências Médicas, Universidade do Estado do Rio de Janeiro, Rio de Janeiro, Brazil; ^2^ Laboratório de Bioquímica de Tripanossomatídeos, Fundação Oswaldo Cruz, Rio de Janeiro, Brazil; ^3^ Laboratório de Imunofisiologia do Exercício, Disciplina de Parasitologia/Faculdade de Ciências Médicas, Universidade do Estado do Rio de Janeiro, Rio de Janeiro, Brazil

**Keywords:** quercetin, *Leishmania braziliensis*, hamsters, reactive species of oxygen, oral treatment

## Abstract

Leishmaniasis is a parasitic disease caused by several species of intracellular protozoa of the genus *Leishmania* that present manifestations ranging from cutaneous ulcers to the fatal visceral form*. Leishmania Viannia braziliensis* is an important species associated with American tegumentary leishmaniasis and the main agent in Brazil, with variable sensitivity to available drugs. The search for new therapeutic alternatives to treat leishmaniasis is an urgent need, especially for endemic countries. Not only is quercetin well known for its antioxidant activity in radical scavenging but also several other biological effects are described, including anti-inflammatory, antimicrobial, and pro-oxidant activities. This study aimed to investigate the flavonoid quercetin’s therapeutic potential in *L. (V.) braziliensis* infection. Quercetin showed antiamastigote (IC_50_ of 21 ± 2.5 µM) and antipromastigote (25 ± 0.7 µM) activities and a selectivity index of 22. The treatment of uninfected or *L. (V.) braziliensis*–infected macrophages with quercetin increased reactive oxygen species (ROS)/H_2_0_2_ generation without altering Nitric Oxide (NO) production. Oral treatment with quercetin of infected hamsters, starting at 1 week of infection for 8 weeks, reduced the lesion thickness (p > 0.01) and parasite load (p > 0.001). The results of this study suggest that the antiamastigote activity of the flavonoid quercetin *in vitro* is associated, at least in part, with the modulation of ROS production by macrophages. The efficacy of oral quercetin treatment in hamsters infected with *L. (V.) braziliensis* was presented for the first time and shows its promising therapeutic potential.

## 1 Introduction

Leishmaniasis is a worldwide parasitic disease caused by several species of intracellular protozoa of the genus *Leishmania* that present with manifestations ranging from skin ulcers to the fatal visceral form. This disease is endemic in approximately 100 countries and modern territories across Europe, Africa, Asia, and the Americas (World Health Organization, 2022). The drug options available for the treatment of the various clinical forms of leishmaniasis are limited, toxic, expensive, and even more critical due to the increasing resistance of the parasites (Amato et al., 2008; Ponte-Sucre et al., 2017; Mendes Roatt et al., 2020). Pentavalent antimonials (Pentostam™ and Glucantime™) are considered the first-choice treatments for leishmaniasis in most affected countries; however, this therapy is associated with a high incidence of adverse effects (Ponte-Sucre et al, 2017). In addition to pentavalent antimonials, other drugs used in the treatment of leishmaniasis as a second choice, such as amphotericin B and pentamidine, are also administered parenterally and cause serious adverse effects that limit and compromise adherence to treatment (Croft and Yardley, 2002; Pradhan et al., 2022). Miltefosine has the advantage of being an orally administered drug, but the sensitivity of *Leishmania* species is variable and its potential teratogenic effect restricts its use (Pradhan et al., 2022).

In addition to the differences in sensitivity naturally existing between the species of *Leishmania* to available drugs, there is also an increase in the reports of the emergence of resistance to these drugs (Ponte-Sucre et al., 2017; Uliana et al., 2018). *Leishmania* resistance mechanisms to conventional treatments involve a number of factors, such as molecular modifications of the parasite such as ATP-binding cassette (ABC) and aquaporin (AQP) transporters, changes in the lipid membrane, and oxidative stress (Ponte-Sucre et al., 2017; Horácio et al., 2021). *Leishmania Viannia braziliensis* is the main etiological agent of American tegumentary leishmaniasis (ATL) and associated with the cutaneous (CL) and mucocutaneous (ML) forms, including in Brazil, with frequent reports of refractoriness to treatment (Amato et al., 2008; Rugani et al., 2018; Anversa et al., 2018). In endemic regions of CL where *Leishmania (V.) braziliensis* is prevalent, the therapeutic failure rate is approximately 50% (Santos et al., 2004; de Prates et al., 2017)

This scenario demonstrates that the development of new drugs is indispensable to leishmaniasis control. The development of therapeutic alternatives for leishmaniasis that can be administered orally has been encouraged in order to facilitate logistics and improve patient adherence to treatment (CONITEC, 2018).

Quercetin is a polyphenolic flavonoid found in a wide variety of foods including citrus fruits, green leafy vegetables, and green tea. In addition to its well-documented antioxidant action (Behling et al., 2004; Gardi et al., 2015; Xu et al., 2019; Song et al., 2020), quercetin has pro-oxidative properties, depending on the used model cells, promoting cytotoxicity to malignant cell lines and embryonic stem cells and in injured neurons (Sak, 2014; Kim and Park, 2016; Bidian et al., 2020; Zubčić et al., 2020). Several therapeutic properties are described for quercetin such as antihypertensive (Marunaka et al., 2017; Elbarbry et al., 2020), anti-inflammatory (Lin et al., 2017; Sato and Mukai, 2020), antiallergic (Kahraman et al., 2003; Sakai-Kashiwabara et al., 2011; Mlcek et al., 2016; Jafarinia et al., 2020), antimicrobial (Giteru et al., 2015; Benjamin et al., 2017; Abbaszadeh et al., 2020), and antiviral activities, including SARS-CoV-2 (Colunga Biancatelli et al., 2020; Derosa et al., 2020). The *in vitro* antileishmanial action of quercetin was previously reported for *L. amazonensis* (Muzitano et al., 2006; Fonseca-Silva et al., 2013; Sousa-Batista et al., 2017) and *L. donovani* (Sen et al., 2008; Mehwish et al., 2021). The quercetin toxic effect on *L. amazonensis* was related to increased reactive oxygen species (ROS) production and mitochondrial dysfunction (Fonseca-Silva et al., 2011), and it is extended to the promastigotes of *L. (V.) braziliensis* (Cataneo et al., 2019). Quercetin also targets arginase in *L. amazonensis* (revised by Carter et al., 2021). However, the therapeutic activity of quercetin in animals infected with *L. (V). braziliensis* has not yet been demonstrated. In this study, our main objective was to evaluate the therapeutic potential of quercetin administered orally in hamsters infected with *L. (V.) braziliensis*.

## 2 Material and methods

### 2.1 Quercetin

The flavonoid quercetin (3, 3´, 4´, 5, 7-pentahydroxyflavone) was commercially obtained (Sigma–Aldrich, St. Louis, MO, USA) and dissolved in dimethylsulfoxide (DMSO, Sigma Aldrich, St Louis, MO, USA). The final concentration of DMSO did not exceed 1% in the cell culture.

### 2.2 Parasites

*L. (V.) braziliensis* (MCAN/BR/98/R619) was routinely isolated from hamsters’ skin lesions. The animals were infected on the dorsal hind paw with 5 × 10^6^ promastigotes of *L. (V.) braziliensis* at the stationary phase. The infection was maintained for 30–40 days, and the skin of the lesion was surgically removed and homogenized with 1 ml of Phosphate-buffered saline (PBS) using a tissue grinder. The cell suspension was incubated with Schneider’s medium (Sigma-Aldrich) containing 20% inactivated fetal bovine serum (FBS) at 27°C. Promastigotes were maintained with weekly passages in Schneider’s medium with 20% FBS and 100 µg/ml gentamicin (Schering-Plough, Kenilworth, New Jersey, USA) at 27°C. Parasites were used for up to five passages in culture, at which time they were reisolated from infected hamsters.

### 2.3 Ethics statement

This study was carried out in strict accordance with the recommendations in the Guide for the Care and Use of Laboratory Animals of the Brazilian National Council of Animal Experimentation. This study protocol was approved by the Ethics Committee on Animal Use of the Instituto de Biologia Roberto Alcantara Gomes of the Universidade do Estado do Rio de Janeiro, by the number protocol 046/2017.

### 2.4 Animals

Female or male golden hamsters (*Mesocricetus auratus*) 6–8 weeks old were obtained from Centro de Criação de Animais de Laboratório (Fundação Oswaldo Cruz, Rio de Janeiro) and maintained under controlled temperature and food and water *ad libitum*.

### 2.5 Antipromastigote activity

The promastigotes of *L. (V.) braziliensis* (5 × 10^5^ cells/well) were cultured in Schneider’s medium supplemented with 20% FBS in the absence or presence of different quercetin concentrations in triplicate (5–320 µM) for 96 h at 27°C. The reference drug miltefosine was used as a positive control at 6 µM. The number of promastigotes was counted daily in a Neubauer chamber.

### 2.6 Macrophage toxicity

To assess the toxicity of quercetin on mammalian cells, resident macrophages were obtained from golden hamsters by peritoneal lavage with 10 ml of a cold RPMI 1640 medium. Cells were plated (4 × 10^6^ in 200 µl) for 1 h at 37°C in the presence of 5% CO_2_, and then, non-adherent cells were removed. Macrophage monolayers were treated in triplicate with quercetin (0–640 µM) for 48 h at 37°C/5% CO_2_. Controls were macrophage monolayers treated with RPMI or 1% of vehicle DMSO (the major final concentration), and the positive control for reduced cellular viability (disrupted cells) was obtained by adding 1% Triton X-100. The viability of macrophages was then assessed by measuring the mitochondrial-dependent reduction of MTT [3-(4, 5-dimethyl- 2-thiazol)-2, 5-diphenyl-2H-tetrazolium bromide)] to formazan. MTT (10 µl to 10 mg/ml) was added to cell cultures and incubated at 37°C/5% CO_2_ for 3 h. The medium was removed, and formazan crystals were dissolved in 180 µl of DMSO. The absorbance was read at 570 nm using a microplate spectrophotometer (µQuant, Biotek Instruments, Inc.). The 50% cytotoxic concentration (CC_50_) was determined by logarithmic regression analysis using GraphPad Prism 6 software.

### 2.7 Antiamastigote activity

The hamster peritoneal cells (2 × 10^6^/ml), obtained as described in 2.6, were plated onto glass coverslips placed within the wells of a 24-well culture plate (0.5 ml/well) and incubated at 37°C/5% CO_2_ for 24 h. After removing the non-adherent cells, the monolayers were infected with *L. (V.) braziliensis* promastigotes (5:1 ratio) for 4 h. The non-internalized parasites were removed, and the infected macrophage monolayers were incubated in triplicate with quercetin (0–320 µM) for 48 h. Controls were incubated with a medium or medium plus vehicle (DMSO 0.02%) or 3 µM miltefosine. After this time, the monolayers were stained with Giemsa, and at least 200 macrophages per sample were counted under optical microscopy. The results were shown as infection index (= % infected macrophages × number of amastigotes/total number of macrophages). The half-maximal inhibitory concentration (IC_50_) was determined by logarithmic regression analysis using GraphPad Prism 6 software.

### 2.8 Measurement of reactive oxygen species production by macrophage

Intracellular levels of ROS in uninfected macrophages or *L. (V.) braziliensis*–infected macrophages were performed using the cell-permeable dye H2DCFDA (2´, 7´-dichlorodihydrofluorescein diacetate). The monolayers of peritoneal macrophages were obtained as described in item 2.6 and plated in a 96-well plate (at 2 × 10^6^/well) and infected with *L. (V.) braziliensis* promastigotes (5:1 ratio) for 4 h. Uninfected or *L. (V.) braziliensis*–infected macrophages were treated with 160 or 320 µM of quercetin for 48 h at 37°C/5% CO_2_. The macrophage monolayers were washed twice with PBS and incubated with 20 mM of H2DCFDA for 30 min at 37°C. Fluorescence was measured in a fluorometer with an excitation wavelength of 507 nm and an emission wave of 530 nm. The positive control was obtained by the addition of 20 units/ml glucose oxidase + 60 mM glucose for 20 min. To evaluate hydrogen peroxide production, the Amplex Red probe (Invitrogen Molecular Probes, Leiden, the Netherlands) was used, following the manufacturer’s recommendations. After quercetin treatment was completed, the wells were washed twice with PBS, the plate was incubated for 30 min with Amplex Red, and the reading was done on a fluorometer with an excitation wavelength of 560 nm and an emission wave of 590 nm. The results were expressed as folds relative to the control (macrophages treated with medium). Each test was performed in triplicate and repeated at least three times.

### 2.9 Evaluation of nitric oxide production

Nitric oxide was measured by detecting nitrite using the Griess reagent. After the respective treatments for 48 h, the supernatants of macrophage monolayers were transferred to the plate where the Griess reagent [1% sulfanilamide added to 0.1% of N-1-naphthylethylenediamine dihydrochloride (Sigma-Aldrich) and 2.5% of phosphoric acid (Sigma-Aldrich)] was added at a ratio of 1:1 (v/v) and incubated for 10 min, at room temperature. Then, the plate was read in an ELISA reader, at 570 nm. The values of the readings were compared with a standard curve of NaNO_2_ (Sigma-Aldrich).

### 2.10 Effect of treatment on infected hamsters

Female or male golden hamsters (8 weeks old) were infected on the dorsal hind paw with 5 × 10^6^ promastigotes of *L. (V.) braziliensis* at the stationary phase. The animals were divided into groups (six-to-eight hamsters per group) 7 days after infection and treated for 8 weeks. The quercetin group (n = 8) was orally treated once a day for five consecutive days a week (2-day treatment-free interval between weeks) with 500 µl of quercetin (20 mg/kg) in an Ora-Plus suspension vehicle (Perrigo^®^, Paddock Laboratories, Minnesota, USA), using an 18G gavage needle (Kent Scientific, Torrington, Connecticut, USA). Control groups were constituted by the untreated group (n = 8) and by the group treated with the reference drug meglumine antimoniate (Glucantime, 80 mg/kg) (n = 6) intraperitoneally (100 µl) three times a week (every other day). The dose of 80 mg/kg of Glucantime was selected based on the range used in previously published studies (Sinagra et al., 2007; Costa et al., 2014; Kawakami et al., 2021) and which proved to be effective in our experimental model. The lesion thickness was measured weekly with a dial caliper (Mitutoyo, Brazil). The animals were euthanized by anesthetic overdose (association of 240 mg/kg of ketamine and 30 mg/kg of xylazine, corresponding to three times the usual anesthetic dose) followed by cardiac puncture for blood collection. Hepatotoxicity and nephrotoxicity were evaluated by the serum dosage of aspartate transaminase (AST), alanine transaminase (ALT), and creatinine, which was performed by the Animal Clinical Analysis Center of the Institute of Science and Technology in Biomodels (Fiocruz RJ) using the Vitros 250 equipment (Orthopedic Clinic—Johnson & Johnson). To determine the parasite load, limiting dilution analysis was used (Costa et al., 2014). The skin of the dorsal infected hind paw and draining lymph node were surgically removed, weighed, and individually homogenized with 1 ml of PBS using a tissue grinder. The cell suspension was serially diluted in quadruplicate (1:10) in Schneider’s medium containing 20% FBS and 100 µg/ml gentamicin at 27°C. The presence of motile parasites was assessed, and the parasite load was determined by the highest dilution in which promastigotes grew after 7–10 days.

### 2.11 Statistical analyses

The data were analyzed by applying one-way analysis of variance with Tukey *post-test* using the GraphPad Prism 6 software program (San Diego, CA, USA). The difference between groups was considered significant when *p* ≤ 0.05.

## 3 Results

### 3.1 *In vitro* activity of quercetin on *L. (V.) braziliensis* promastigotes and intracellular amastigotes

**Figure 1:**
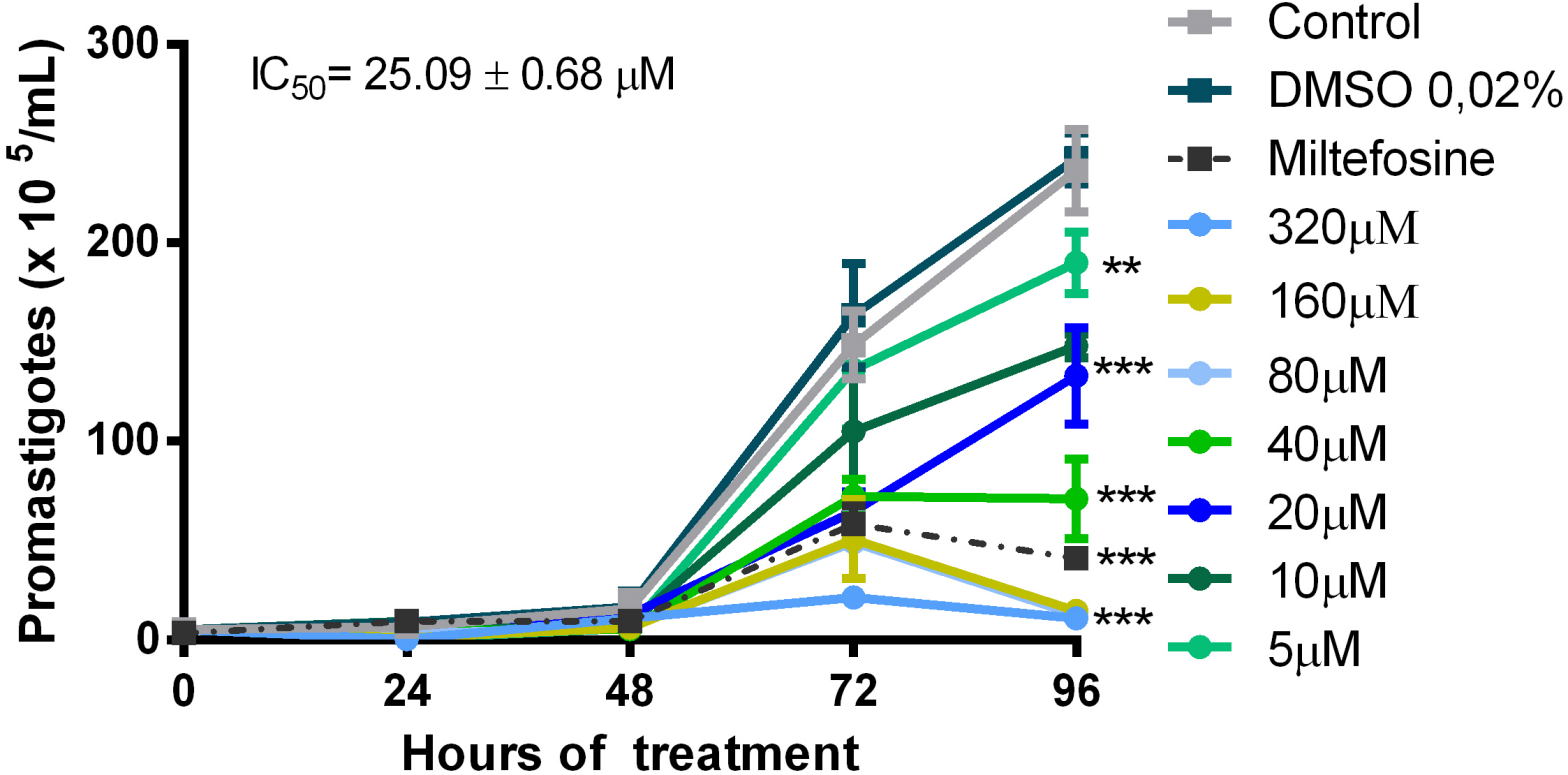
Activity of quercetin on the promastigote of *L. (V.) brazilliensis*. Promastigotes were cultivated in Schneider’s medium supplemented with 20% fetal bovine serum (FBS) at 27°C for 96 h, in the absence or presence of quercetin (indicated concentrations). The number of parasites was determined daily by counting in a Neubauer chamber. Controls were promastigotes cultured with a vehicle [0.02% dimethylsulfoxide (DMSO)] or 6 µM miltefosine as a reference drug. The data presented are representative of three independent experiments performed in triplicate. Mean ± SD, n = 3. **p < 0.01, ***p < 0.001 (difference compared to DMSO or medium).

**Figure 2:**
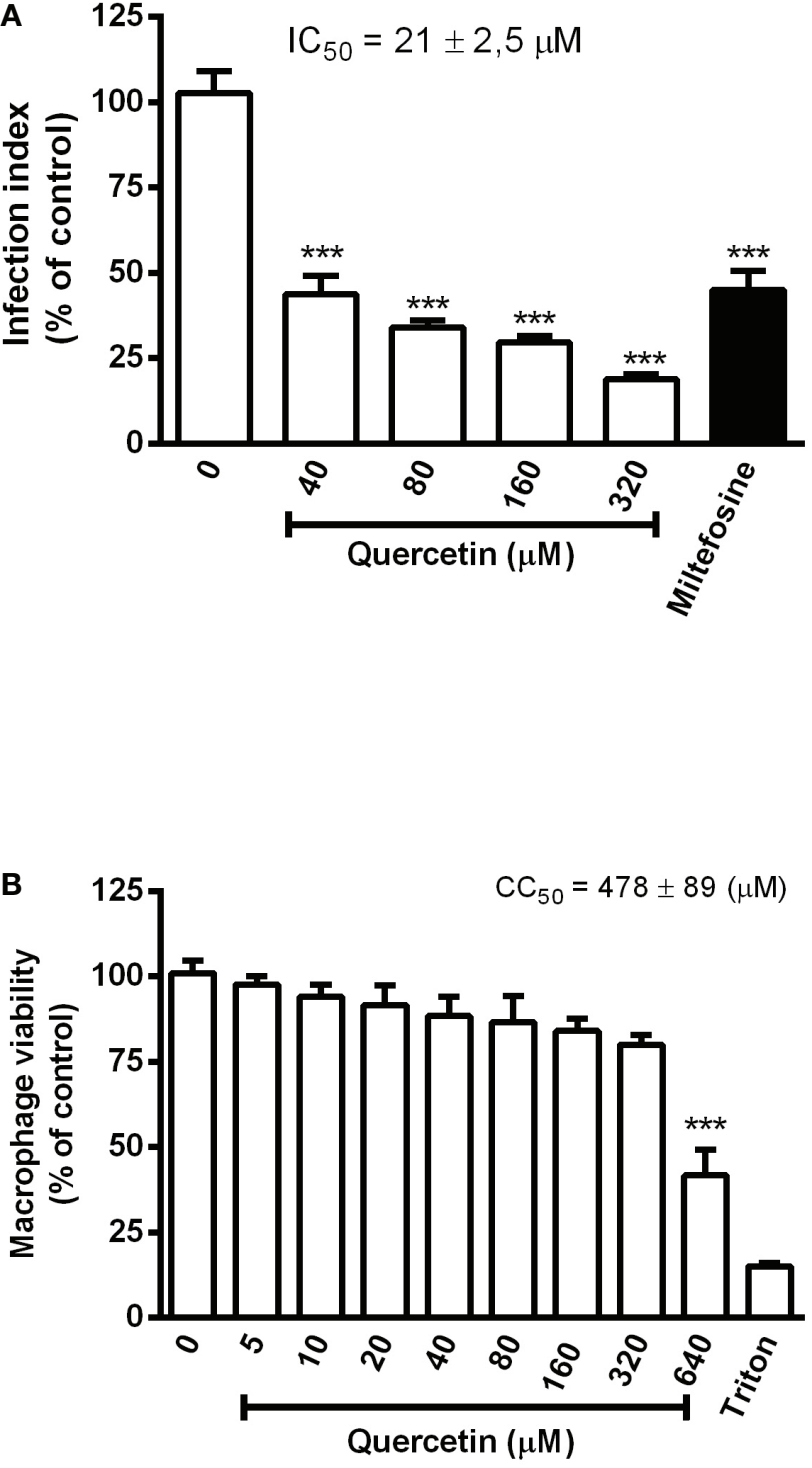
Effect of quercetin on the intracellular amastigotes of *L. (V.) braziliensis* and macrophage toxicity. **(A)** Monolayers of hamster peritoneal macrophages infected with *L. (V.) braziliensis* (5:1 ratio) were treated with the indicated concentrations of quercetin for 48 h. Controls were treated with a 0.02% DMSO vehicle or 3 µM miltefosine. After treatment, macrophage monolayers were stained with Giemsa and the infection index was established by counting at least 200 cells on each coverslip in triplicate. **(B)** Hamster peritoneal macrophage monolayers were incubated in triplicate with quercetin for 48 h, and cell viability was measured using the 3-(4, 5-dimethyl- 2-thiazol)-2, 5-diphenyl-2H-tetrazolium bromide) assay. Controls were vehicle (DMSO) or 0.1% Triton X-100 as a positive toxicity control for reduced cell viability. Values presented represent the mean ± SD of three independent experiments and are expressed as a percentage of control. ***p < 0.001 (difference compared to DMSO).

For the *in vitro* evaluation of quercetin activity against *L. (V.) braziliensis*, we performed analyses on the promastigotes and intracellular amastigotes. Promastigotes were cultured with quercetin (0–320 µM) or miltefosine (reference drug) for 96 h, and their growth was evaluated by daily counting. Quercetin was able to significantly reduce the growth of promastigotes in a dose-dependent manner (**Figure 1**), and the IC_50_ was estimated at 25 ± 0.68 µM (96 h). The activity against intracellular amastigotes was evaluated using macrophage monolayers infected with *L. (V.) braziliensis* and treated with quercetin (0–320 µM) or miltefosine (IC_50_) for 48 h. In a dose-dependent manner, quercetin was able to significantly decrease the number of amastigotes in macrophages, with an IC_50_ estimated at 21 ± 2.5 µM (**Figure 2A**). As expected, the reference drug miltefosine, used at the concentration relative to the IC_50_, was able to reduce the number of intracellular amastigotes.

The toxicity of quercetin to hamster macrophages was evaluated after 48 h of treatment. Cells showed a significant loss of viability only from 640 µM onward, with an estimated CC_50_ of 478 ± 89 µM (**Figure 2B**). The selectivity index (CC_50_/IC_50_) of quercetin was calculated to be 22, meaning that it is 22 times more toxic to intracellular amastigotes of *L.(V.) braziliensis* than to hamster macrophages.

### 3.2 Quercetin induces ROS without altering NO production by macrophages

**Figure 3:**
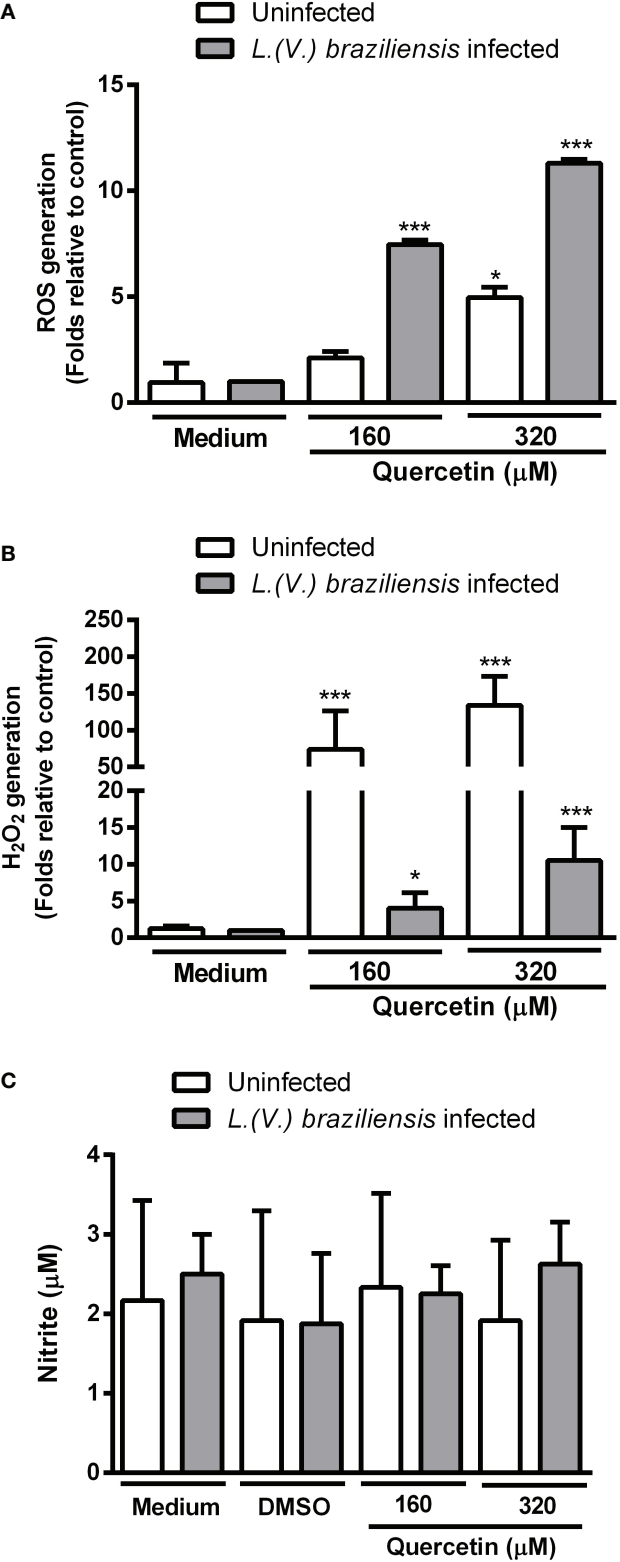
Production of toxic radicals by macrophages. Monolayers of peritoneal macrophages were infected or not with *L. (V.) braziliensis* and incubated in the presence or absence of quercetin for 48 h. **(A)** Reactive oxygen species (ROS) generation was measured using the fluorescent probe 2´, 7´-dichlorodihydrofluorescein diacetate, **(B)** H_2_O_2_ was measured by the *Amplexred* probe. Data were expressed as a fold increase in ROS production relative to control. **(C)** Nitric oxide production was evaluated by the Griess method and the results expressed as nitrite concentration. The values shown represent the mean ± SD of three independent experiments. *p < 0.05, ***p <0.001 (difference compared to DMSO or medium).

To investigate whether quercetin anti-amastigote activity was associated with the ability to modulate host cells, we evaluated ROS and NO production by infected and uninfected macrophages. After 48 h of treatment with quercetin at 160 or 320 µM, intracellular ROS was evaluated by the H2DCFDA probe and NO was analyzed in the culture supernatants by the Griess method. In **Figure 3A**, we show a significant increase in ROS production by the infected macrophages treated with quercetin in both concentrations and in uninfected macrophages at the highest concentration of quercetin tested (320 µM). Using the Amplex Red^®^ probe, it was possible to observe that treatment with quercetin significantly increased the production of H_2_O_2_ in infected and uninfected macrophages (**Figure 3B**). In uninfected macrophages, the increase in H_2_O_2_ was more than 70- and 100-fold with treatment at 160 and 320 µM of quercetin, respectively. In relation to NO, we observed no change in nitrite production in either *L. (V.) braziliensis*–infected or –uninfected macrophages treated with quercetin for 48 h (**Figure 3C**).

### 3.3 Therapeutic activity of quercetin in hamsters infected with *L. (V.) braziliensis*

**Figure 4:**
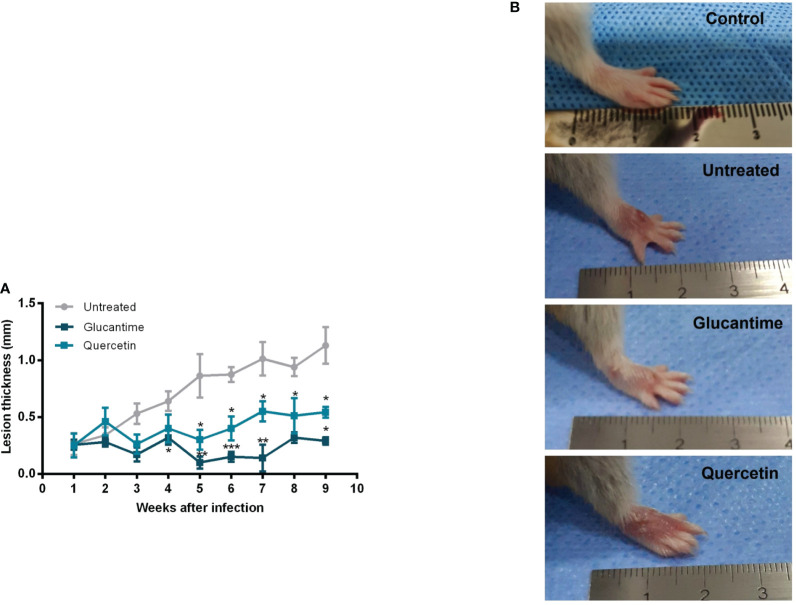
Therapeutic effect of quercetin by the oral route on hamster infected with *L. (V.) braziliensis*. Hamsters (six-to-eight animals per group) were infected in the dorsal hindpaw with 5 × 10^6^ promastigotes of *L. (V.) braziliensis* and treated from the seventh day of infection for eight weeks with oral quercetin (20 mg/kg; five times a week) or glucantime (80 mg/kg; three times a week) intraperitoneally. **(A)** The thickness of the lesions was measured weekly and expressed as mean + standard error. **(B)** Representative images of the lesion of the animals of each experimental group and of an uninfected animal for reference. These results are representative of two independent experiments. *p < 0.05, **p < 0.01, and ***p < 0.001 (difference compared to the untreated group).

**Figure 5:**
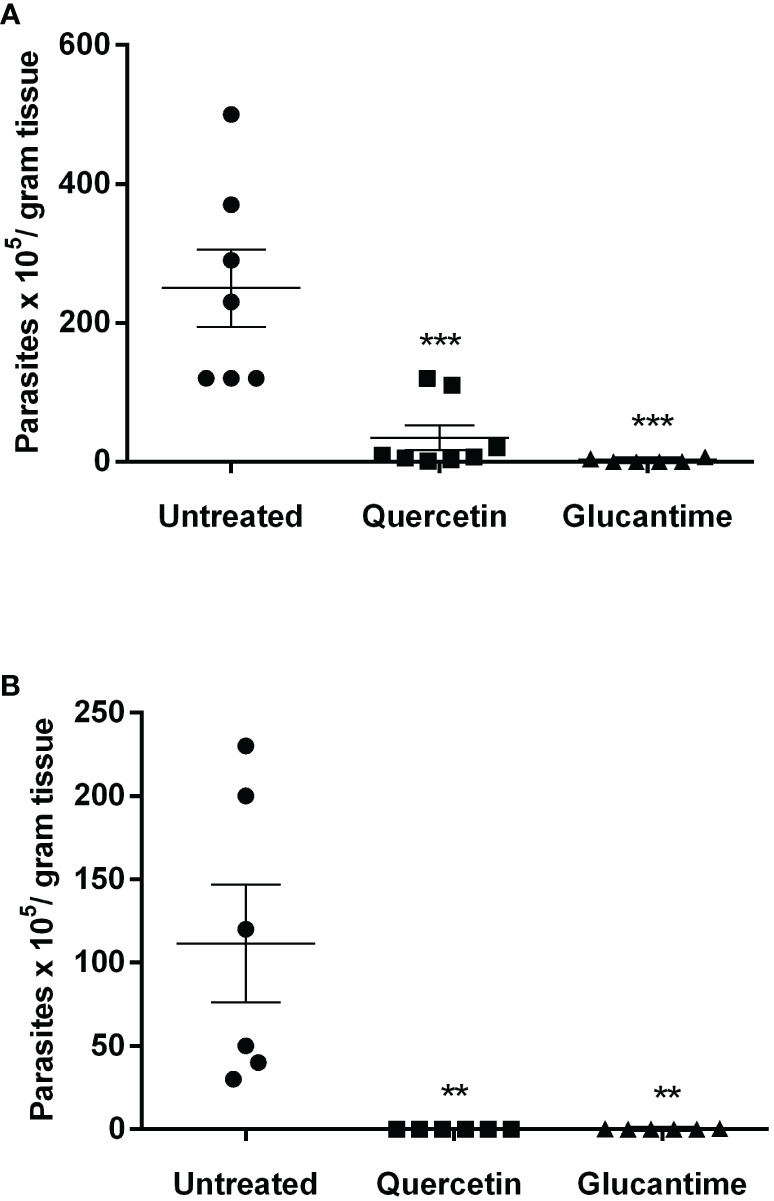
Parasitic load in the lesion and draining lymph nodes. The animals were euthanized, and the parasitic load was determined by the limiting dilution assay in the paw lesion **(A)** and the draining lymph nodes **(B)** 1 week after the end of treatment (9 weeks of infection). Each point represents one animal, and the horizontal bars express the mean values. The data are representative of two independent experiments. **p < 0.01, and ***p < 0.001 (difference compared to the untreated group).

**Figure 6:**
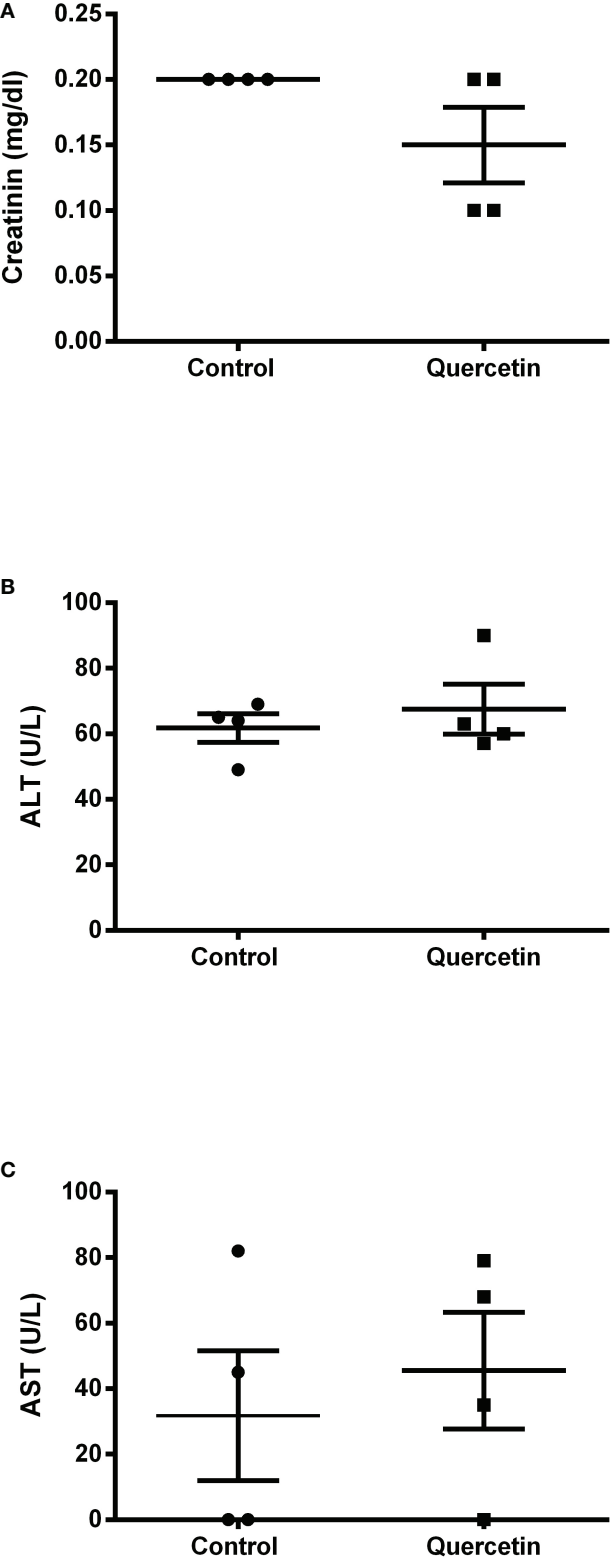
Serum toxicological analysis. At the end of treatment, serum samples were collected from the animals (n = 4) for the colorimetric determination of creatinine **(A)**, alanine transaminase **(B)** and aspartate transaminase **(C)** concentrations, as toxicity parameters for the liver and kidneys. Each point represents one animal, and the horizontal bars express the mean values.

Using the susceptible experimental model for infection with *L. (V.) braziliensis*, the golden hamster, we evaluated the action of orally administered quercetin (20 mg/kg). Treatments with oral quercetin (five times a week) or intraperitoneal Glucantime (three times a week) were started from 7 days of infection (lesion average thickness = 0. 26 mm) and maintained for 8 weeks (9 weeks of infection). Quercetin treatment was able to significantly control lesion thickness from the fourth week of treatment (**Figure 4**) when compared to the untreated group. As expected, the reference drug Glucantime significantly controlled the lesion development. At the ninth week of infection, the lesions of the untreated animals were ulcerated, besides being smaller than untreated group, no ulceration was observed in the lesions of the animals treated with quercetin (**Figure 4B**). The results showed that the treatment with quercetin reduced significantly parasitic load in both the lesion (**Figure 5A**) and the draining lymph node (**Figure 5B**). Treatment with quercetin for 8 weeks did not alter the levels of renal (creatinine) and hepatic (ALT and AST) toxicity parameters, which were similar when compared to untreated animals (**Figure 6**).

## 4 Discussion

The treatment of CL continues to be a challenge, and there is an urgent need to discover new efficient and safe active drugs, particularly for local or oral use, that increase patient compliance. In the present study, for the first time, the therapeutic effect of the oral flavonoid quercetin was demonstrated in hamsters infected with *L. (V.) braziliensis*, the main species causing ATL, especially in Brazil.

*In vitro*, we showed that quercetin has activity against both promastigote (IC_50_ = 25 ± 0.7 µM/96 h) and intracellular-amastigote (IC_50_ = 21 ± 2.5 µM/48h) forms of *L. (V.) braziliensis*. Some studies have already reported *in vitro* the activity of quercetin for some species of Cataneo and collaborators (2021) showed which quercetin at 48 and 70 µM reduced the number of intracellular amastigotes of *L. (V.) braziliensis* within 24 h of treatment; however, they did not determine the IC_50_. In another study, quercetin reduced the promastigote growth of *L. (V.) braziliensis* and *L. (V.) panamensis*, with IC_50_ estimated at 30 and 60 µM (72 h), respectively (Marín et al., 2009). Quercetin showed activity on *L. amazonensis* and IC_50_ determined at 3.4 µM (72 h) for intracellular amastigotes (Fonseca-Silva et al., 2013) and 31.4 µM (48 h) for promastigotes (Fonseca-Silva et al., 2011). Mehwish and collaborators (2021) demonstrated that quercetin has antiamastigote activity against *L. donovani* with an IC_50_ of 240 µM (72 h). The differences found in the IC_50_ of quercetin may reflect the variations in the sensitivity of each *Leishmania* species, as well as the experimental protocol, especially the duration of treatment. Although we have not investigated the antiparasitic mechanisms of quercetin directly in the parasite, it is possible that quercetin induces the generation of ROS and disrupts the parasite’s mitochondria as seen for *L. amazonensis* (Fonseca-Silva et al., 2011).

In our results, the quercetin CC_50_ for hamster macrophages was estimated at 478 ± 89 µM in 48 h of treatment. This result differs slightly from the findings of other studies with different cell types and treatment times. For example, studies performed in J774 macrophages treated for 72 h with quercetin had the CC_50_ estimated at 125 µM (Marín et al., 2009), while, for mice peritoneal macrophages, the IC_50_ was 80 µM (Fonseca-Silva et al., 2013).

In order to determine whether the antiamastigote activity of quercetin would involve the modulation of host cell microbicidal activity, we evaluated the macrophage production of ROS and NO. In our results, we observed that quercetin did not induce changes in NO production; however, it increased ROS by both infected and uninfected macrophages. These results suggest, at least in part, which activity of quercetin against *L. (V.) braziliensis* involves the stimulation of ROS production by macrophages. Our findings are in agreement with studies on macrophages infected with *L. amazonensis* that demonstrated that quercetin induces an increase in ROS production by infected macrophages (Fonseca-Silva et al., 2013). In macrophages infected with *L. (V.) braziliensis*, quercetin reduced the number of amastigotes without modulating NO production, although it reduced alpha tumor necrosis factor- alfa (TNF-α) levels and increased IL-10 synthesis (Cataneo et al., 2019). The authors showed that treatment with quercetin was able to decrease labile iron in macrophages through the regulation of Nrf2/HO-1 expression, resulting in a decrease in the iron available to the parasite and consequently inducing its death (Cataneo et al., 2019).

Although the production of toxic radicals by macrophages (such as ROS and NO) is crucial for the control of infection by intracellular microorganisms, such as *Leishmania*, the excess of these mediators is also associated with tissue damage and pathogenesis (revised by Bogdan, 2020; Reverte et al., 2022) Several studies have demonstrated the anti-inflammatory effects of quercetin involving the inhibition of nitric oxide production as well as the production of proinflammatory cytokines (Kim et al., 2004; Tsai et al., 2022). In this sense, quercetin appears to be an interesting drug candidate for leishmaniasis since it has both direct antileishmanial activity and the potential to modulate the microbicidal and inflammatory activity of macrophages.

In the present study, we demonstrated the therapeutic effect of oral quercetin in hamsters infected with *L.(V.) braziliensis*. The hamster model is sensitive to infection by *L.(V.) braziliensis*, developing a pattern of cutaneous leishmaniasis that resembles the human disease; therefore, it is useful for therapeutic and vaccine trials (Gomes-Silva et al., 2013; Loría-Cervera and Andrade-Narváez, 2014; Ribeiro-romão et al., 2014, Mears et al., 2015).

We showed that the treatment with oral quercetin of hamsters infected with *L.(V.) braziliensis* from 7 days of infection, when the lesion was at the beginning (0.26 mm), significantly controlled the lesion size, as well as reduced the parasite load both in the lesion and in the draining lymph node. Despite the *in vitro* demonstration of the antileishmanial action of quercetin for several species of the parasite, there are relatively few studies conducted *in vivo* to investigate its therapeutic activity in experimental leishmaniasis. However, our *in vivo* findings demonstrating the potential of quercetin in experimental cutaneous leishmaniasis corroborate previous studies in both cutaneous and visceral leishmaniasis models. Quercetin administered orally (16 mg/kg of body weight) from 7 days of infection for 30 days controlled the lesion and reduced the parasite load in BALB/c mice infected with *L. amazonensis* Muzitano et al., 2009). In a study conducted in hamsters infected with *L. donovani*, quercetin administered orally reduced the number of parasites in the spleen. Another study demonstrated that the encapsulation of quercetin in the lipid nanocapsules (LNCs) of poly(ϵ-caprolactone) was able to increase its efficacy in the treatment of BALB/c mice infected by *L. amazonensis* (Sousa-Batista et al., 2017).

In conclusion, the present study demonstrated for the first time the effect of quercetin in hamsters infected with *L. (V.) braziliensis*. Furthermore, the antileishmanial activity of quercetin may be associated with not only a direct action against the parasite but also the modulation of ROS production by macrophages.

## Data availability statement

The original contributions presented in the study are included in the article/supplementary material. Further inquiries can be directed to the corresponding author.

## Ethics statement

The animal study was reviewed and approved by Ethics Committee for the Care and Use of Experimental Animals- Instituto de Biologia Roberto Alcantara Gomes- Universidade do Estado do Rio de Janeiro.

## Author contributions

RFS conducted experiments and contributed to the writing of the manuscript. TS, ACSB, JDI, BDV, MAPM, BFA, LMS contributed the experiments. EEAA and JDI contributed to the execution and the discussion of ROS analysis. PMLD contributed to data discussion and manuscript writing. SAGS supervised the experiments, analyzed the results and wrote the manuscript.
